# The Principle of Inversion: Why the Quantitative-Empirical Paradigm Cannot Serve as a Unifying Basis for Psychology as an Academic Discipline

**DOI:** 10.3389/fpsyg.2020.596425

**Published:** 2020-12-07

**Authors:** Roland Mayrhofer, Fabian Hutmacher

**Affiliations:** ^1^Department of Psychology, University of Regensburg, Regensburg, Germany; ^2^Human-Computer-Media Institute, University of Würzburg, Würzburg, Germany

**Keywords:** self-perception of psychology, principle of inversion, methods in psychology, operationalism, definitions of psychology

## Introduction

In the English-speaking world, as well as in the international academic discourse and many other languages, the term “science” or appropriate translations refer only to a certain area of knowledge, namely the natural and the social sciences, thus excluding what is usually referred to as humanities or *Geisteswissenschaften* (Szostak, 2004).

The history and philosophy of science shows that the sciences rely heavily on the so-called Scientific Method, a set of theoretical and methodological principles which consists, in essence, of observing, formulating hypotheses and testing these hypotheses in experiments, in order to discover general laws. In doing so, knowledge is generated by relying on empirical evidence, which in turn expresses directly observable phenomena in terms of quantitative data. Excluding the extremely complex discussions about advantages, drawbacks, and alternatives to the Scientific Method (e.g., Gower, 1997; Nola and Sankey, 2014; Andersen and Hepburn, 2015), it is important to note that quantitative-empirical methods and thinking according to the Scientific Method dominate the sciences (Haig, 2014; Nola and Sankey, 2014; Sankey, 2014) and also psychology (Garber, 2019; Haig, 2019; Toomela, 2020).

By contrast, the humanities rely much less on quantitative—let alone experimental—methods, although these are employed nonetheless when appropriate. Instead of producing and gathering empirical data, the humanities characteristically approach their subject matter from a descriptive, interpretive, and hermeneutical understanding whose historical and comparative angles cannot be conveniently summarized by a single term (Watanabe, 2010; Bem and De Jong, 2013; Leezenberg and De Vries, 2019).

## Psychology as an Academic Discipline and the Principle of Inversion

Psychology is usually portrayed as the study of the human mind and behavior, although this nomenclature does not make it entirely clear what it actually designates.

In ancient Greek, “*psyche*” encompasses a variety of meanings (Claus, 1983) such as stream of air, breath of life, substance of life (in an ontological sense), spirit, mind, soul, personality, consciousness, self, or even ghost (of the dead). Although these terms describe a semantic field with the underlying term “life,” this does not tell us what psychologists actually investigate. The term “psychology,” in the sense of “study of the *soul*,” originated in the early modern era and was employed mostly for topics which would be categorized to be part of philosophy today. Nevertheless, the founders of modern academic psychology with its predominantly experimental and empirical orientation in the nineteenth century—most notably Wilhelm Wundt—continued to use this term. Further historical research (such as Russo Krauss, 2019) might explain why “*psyche*” was retained as term for their subject matter, thus clarifying the conceptual ideas behind actual research.

This heterogeneity of “*psyche*” is mirrored in the different psychological subdisciplines, such as cognitive, social, or biological psychology and the many branches of applied psychology, such as educational, organizational, or clinical psychology. And while mind, personality, consciousness, and self are familiar terms in psychology, it is clear that other aspects of the Greek “*psyche*” such as the physical properties of breath are not part of the discipline.

In short, psychology investigates many aspects of human existence—but then how does psychology differ from, say, anthropology, sociology, or history? Wherein lies the unity of psychology as academic discipline?

A widely-used textbook (Gerrig, 2012, p. 2) gives the following answer: Many psychologists seek answers to the fundamental question: “What is human nature?” This question is pursued by looking at processes that occur within individuals, and thus psychology is defined as the scientific study of the behavior of individuals and their mental processes (see also e.g., Lilienfeld et al., 2015; Myers and Dewall, 2015 for similar conceptualizations). However, the main elements of this train of thought—human nature, the individual in its entirety, and a scientific approach—reveal that it is not a trivial matter to state precisely what psychology is actually about.

First, it seems debatable that Gerrig's attempt to subsume the subject matter of psychology under the umbrella term “human nature” is really more precise than hazy concepts such as “*psyche*” or “soul” which contemporary psychology has dismissed as too vague (Haaga, 2004; Henriques, 2004; Lilienfeld, 2004). Whereas academic psychologists might argue that they are not interested in such a hazy concept but rather in specific topics such as emotions, neurobiology, or education, all these concepts revolve around the human mind and behavior. Therefore, psychology does indeed have some kind of common theme or center—but this center is so vague that it cannot act as focal point or provide the same clear framework as the subject matters of other disciplines. By contrast, physics is also very diverse, possibly even more so than psychology, but its subject matter is clearly defined as matter and the related phenomena of energy, space and time.

Second, Gerrig's assumption that the answer to the question about “human nature” can be found “by looking at processes that occur within individuals” is not self-evident. Simply put, focusing on the individual is problematic because many—if not all—of the individuals' intrinsic processes are inextricably intertwined with larger social, societal, or historical contexts (e.g., Agassi, 1977; Margolis, 1995, 2008). In other words, the behavior of individuals and their mental processes are shaped by outside contextual and societal factors, which may vary over time. The failure to take variability into consideration might underestimate the complexity of mental processes and give the impression that “human nature” is more hard-wired and less context-dependent than it actually is.

All of this shows that the exact subject matter of psychology is hard to pinpoint or to distinguish from other disciplines which also deal with behavior and mental processes, such as anthropology, history, cultural and literary studies, or philosophy. Nevertheless, Gerrig's definition contains an element which is crucial for the self-conception of psychology as discipline, namely the emphasis on “scientific study.” Similarly, the APA Dictionary of Psychology explicitly emphasizes “observation, experimentation, testing, and analysis” (VandenBos, 2015, p. 860) as characteristic research methods, echoing the Scientific Method.

As early as 1983, Jüttemann pointed out that the common factor underlying the various branches and areas of research in psychology is not characterized *thematically* but rather by a common method and methodology, namely the rather strict—and sometimes dogmatic—adherence to the Scientific Method. He termed this “the principle of inversion”: in other disciplines method and methodology are aligned with the respective subject matter, but in psychology this principle is inverted (see also Royce, 1961; Michell, 1997; Summers, 2012).

Jüttemann's astute observation has two interesting consequences: First, there is a stark contrast between the rather strict methodological requirements and the very broad and often hazy thematic content of psychological research. Therefore, Jüttemann concludes ironically, “everything done by psychologists who employ the nomological methodology in their research must count as academic psychology” (Jüttemann, 1983, p. 34, translated). Second, this modus operandi differs from other academic fields which either have clearly defined subject matters or employ much less rigid methodologies.

The Scientific Method originated long before the institutionalization of psychology as an academic discipline in the late nineteenth century. Moreover, it is closely associated with the natural sciences, especially physics. Thus, psychology is dominated by a method which is neither unique to nor was developed within the own academic field. By contrast, methods which were developed within and specifically for the framework of psychology such as psychoanalysis or introspection are relegated to the fringes of academic psychology.

Jüttemann argues that the rigid methodology has far-reaching consequences for the very nature of psychology and criticizes the resulting “research operationalism,” i.e., the fact that the subject matters of certain areas—such as stress—are only represented by phenomena and procedures which conform to operationalizations according to the Scientific Method. But by reducing complex phenomena to easily quantifiable laboratory procedures the concepts in question—e.g., stress—lose their original meaning. In essence, this means that by solely using the Scientific Method to investigate psychological phenomena (such as stress) we do not learn much about these phenomena as such. Rather, we transform them into something else which can be quantified and measured, meaning that highly complex phenomena are simplified in order to make them quantifiable (see also Hibberd, 2019; Mayrhofer et al., in press). “Stress” is a concrete example because the concept of stress “is deeply intertwined with the constituents of modern identity” (Hutmacher, 2019, p. 181) and therefore extremely complex, although research on stress highly relies on quantifiable parameters.

Whereas this operationalizing of psychological concepts might be appreciated as a more precise specification, it also goes hand in hand with a narrowing of real-life phenomena. It is possible that stripping complex phenomena down to their—supposedly!—bare bones reveals their core mechanisms. But more often we lose important aspects during this process, thereby missing the opportunity to understand something in its entirety. In other words, the professed exactitude and the desire to uncover the fundamental mechanisms of mental and behavioral phenomena by employing a quantitative-empirical paradigm within the very framework of the Scientific Method inadvertently misses important aspects.

To put it differently, it is by no means self-evident that studying internal—mental or behavioral—processes according to a certain predefined method will yield the desired results or that other methods might not provide more or a different kind of insight. Furthermore, even if we gain some knowledge by applying the Scientific Method, it is not an evident conclusion that this will also tell us something about human nature.

## Discussion

Where does this leave academic psychology? Thematically, psychology is a very colorful picture of different subject matters, whose interconnectedness is often rather tenuous and does not display a strong cohesiveness while circling around “human nature” as a hazy center of gravity. However, this vibrant mixture is hidden behind a veneer of uniformity, which manifests itself in the strict adherence to the quantitative-empirical method. This uniformity certainly conveys strength because of its methodological rigor and scientific respectability. However, simultaneously it hampers psychology by preventing it from exploring other avenues which might yield additional insight into mental and behavioral processes or even human nature.

The idea of a one-stop method is problematic for two reasons: First, psychology as a field is wide and diverse. Second, the specific mental and behavioral phenomena—such as stress—are hard to define precisely (Zagaria et al., 2020). Therefore, applying something seemingly precise such as the Scientific Method is inherently at odds with trying to understand such hard-to-grasp, complex phenomena. In short, the quantitative-empirical method cannot serve as a unifying basis for psychology as an academic discipline because it misses important dimensions of “human nature.”

We believe that postmodern approaches, which were specifically developed to describe the complexities and ambiguities of modern societies, may offer a way out of this dilemma, although here we can only give a brief sketch: Postmodern approaches recognize and emphasize that a certain phenomenon may be understood by using different methods. Seemingly different phenomena and/or approaches often point into the same direction, although from different perspectives (e.g., Bertens, 1995; Sim, 2011; Aylesworth, 2015). This does not mean that there is no “truth” in psychology or that we cannot approach this truth (Holtz, 2020). Rather, the strength of a postmodern mindset lies in the ability to describe and to comprehend very complex phenomena without watering them down.

Therefore, such approaches will probably expand both the range and the explanatory power of psychology. As mental and behavioral processes tend to be innately fuzzy, any investigation of these phenomena must take this fuzzy nature into account. This is of course no plea to abandon the quantitative-empirical methods as they have revealed many interesting aspects of the *psyche*. The human mind and behavior are diverse—so why should our methods for investigating them not be equally diverse? Although largely outside the “scientific mainstream,” there are other schools of thought in psychology which operate on the basis of different concepts of science, such as psychoanalysis (Bazan, 2018), humanistic (Warmoth, 1998), constructivist (Lincoln and Hoffman, 2019), or phenomenological (Langdridge, 2007) psychology. Taking their approaches seriously is likely to turn academic psychology into a vibrant generator of relevant knowledge and to spark more light to the enigma we term *psyche*.

## Author Contributions

RM and FH developed the idea for the article. RM drafted the manuscript. FH provided feedback and suggestions. Both authors approved the manuscript for submission.

## Conflict of Interest

The authors declare that the research was conducted in the absence of any commercial or financial relationships that could be construed as a potential conflict of interest.
